# Platform for a Hydrocarbon Exhaust Gas Sensor Utilizing a Pumping Cell and a Conductometric Sensor

**DOI:** 10.3390/s90907498

**Published:** 2009-09-18

**Authors:** Diana Biskupski, Andrea Geupel, Kerstin Wiesner, Maximilian Fleischer, Ralf Moos

**Affiliations:** 1 Bayreuth Engine Research Center, Functional Materials, University of Bayreuth, 95440 Bayreuth, Germany; 2 Siemens AG, Corporate Research & Technology, Sensor Systems, 81739 Munich, Germany

**Keywords:** hydrocarbon exhaust gas sensor, strontium titanate, gallium oxide, electrochemical pumping cell, exhaust gas sensor

## Abstract

Very often, high-temperature operated gas sensors are cross-sensitive to oxygen and/or they cannot be operated in oxygen-deficient (rich) atmospheres. For instance, some metal oxides like Ga_2_O_3_ or doped SrTiO_3_ are excellent materials for conductometric hydrocarbon detection in the rough atmosphere of automotive exhausts, but have to be operated preferably at a constant oxygen concentration. We propose a modular sensor platform that combines a conductometric two-sensor-setup with an electrochemical pumping cell made of YSZ to establish a constant oxygen concentration in the ambient of the conductometric sensor film. In this paper, the platform is introduced, the two-sensor-setup is integrated into this new design, and sensing performance is characterized. Such a platform can be used for other sensor principles as well.

## 1. Introduction

**Figure 1.**
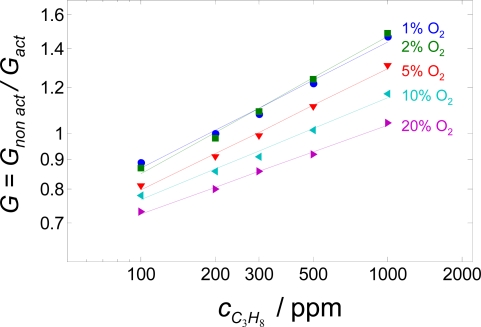
Normalized conductance (non-activated value divided by the activated one of a 1% donor-doped SrTiO_3_. Data recalculated from [12]. *T* = 700 °C.

In order to monitor the air-to-fuel ratio and to detect directly the aging status of exhaust gas aftertreatment systems, ceramic oxygen gas sensors have been serialized [1,2]. For on-board diagnosis (OBD), hydrocarbon (HC) sensors have been proposed [3], mainly based on a mixed potential principle [4,5]. In another approach, it was proposed to detect HC directly in the exhaust with conductometric sensors of metal oxides such as gallium oxide (Ga_2_O_3_) [6] or doped strontium titanate (SrTiO_3_) [7]. Since the resistance of these materials also depends on the oxygen concentration of the exhaust and on the sensor temperature [8,9], a two-sensor-setup was introduced [10,11], with one sensor being catalytically activated, whereas the other one remained non-activated. The activated sensor measures the oxygen concentration in the equilibrium state. It should show the same temperature dependency as the non-activated one. Hence, it acts as a resistive λ-sensor and is not sensitive to HC anymore. The ratio of both sensor signals is expected to result in a sensor signal that is almost unaffected by temperature and oxygen content of the exhaust. However, in a later paper [12] it turned out that e.g., for 1% Ta-doped SrTiO_3_ this method is not sufficient to compensate the oxygen cross-interference completely. The compensation works only effectively at low oxygen concentrations, resulting in a sensor signal that is insensitive to oxygen. In Figure 1, the normalized conductance *G* (ratio of conductance of non-activated and activated sensor) is plotted. The data are recalculated from Figure 5 of Ref. [12]. Obviously, only at low oxygen concentrations an (almost) oxygen independent behavior occurs. At higher oxygen concentrations, *G* depends on the oxygen concentration. The reason for this is that the conductivity of the activated material is almost insensitive to oxygen, whereas the conductivity of the non-activated one also depends on the oxygen concentration [12].

In some older patents, a combination of an oxygen pumping cell with a sensor for combustibles to establish a well-defined oxygen concentration at the conductometric device, even if the oxygen content fluctuates, was suggested [13]. For the first realizations using a thimble-type oxygen sensor mounted onto a steel cell, a commercially available conductometric SnO_2_ sensor was used [14].

In an initial setup, we prepared a *planar* sensor in a multilayer ceramic technology including the challenge to join alumina and zirconia. It was verified that it is possible to establish a constant oxygen concentration at the cavity where the sensor film is located by applying an appropriate pumping voltage to the cell [15]. Due the pumping voltage, oxygen ions are transported through the YSZ membrane, leading to an adjustable oxygen concentration at the position where the gas sensitive film is located.

**Figure 2.**
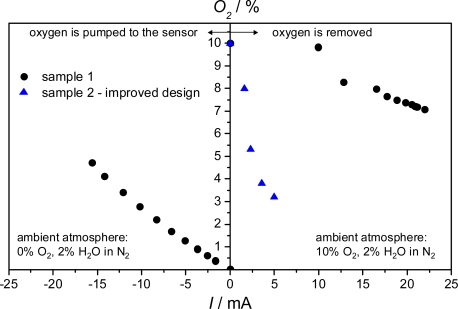
Oxygen concentration at the sensor film versus the pumping current for rich and lean exhaust gas. For rich gas (0% O_2_) the oxygen was delivered to the sensitive film. With a lean gas flow (10% O_2_) oxygen was pumped out of the sensor chamber and the current direction is reverse. Data reassembled from [15]. *T* = 700 °C.

The assembly as described in [15] has two main cavities – a sensor chamber and a pumping chamber, which are connected via a very small diffusion channel. The electrochemical pumping cell presents the ceiling of the pumping chamber with one electrode facing the chamber and the opposed one facing the exhaust gas. The sensor chamber has access to the exhaust gas via a second diffusion channel. Since the highest sensitivity of donor-doped SrTiO_3_ occurs at an oxygen concentration of 5% or less, in an initial approach it was demonstrated that one can establish a defined oxygen concentration by the electrochemical cell. For that purpose, a conductometric oxygen sensing film of SrTi_1−*x*_Fe*_x_*O_3−δ_ was applied instead of a hydrocarbon sensitive film. Details of this oxygen sensor material can be found in ref. [16]. A defined oxygen concentration was set in the ambient gas. At the beginning, no pumping voltage was applied. Then, the pumping voltage was increased stepwise. The pumping current followed immediately the pumping voltage, as well as the oxygen sensor resistance. Using a previously measured correlation between sensor film resistance and oxygen concentration, a relationship between cell pumping current and obtained oxygen concentration at the sensor film can be derived for several ambient oxygen concentrations. In Figure 2, the oxygen concentration at the film is plotted versus the electrical pumping current. Obviously, it is possible to establish a defined oxygen concentration within the requested range at the position of the sensor film independently of the oxygen partial pressure in the exhaust gas. As typical for rich exhausts, no oxygen but water was present in the gas flow regarding the left part of the graph. Oxygen could, however, be generated by electrolyzing H_2_O at the electrode facing the exhaust gas where it is abundant. The required current for obtaining an oxygen concentration at the film is about 15 mA. If 10% O_2_ were present in the exhaust gas, one has to pump out oxygen from the sensor chamber. With sample 1, the oxygen concentration could not be reduced as required due to a leak at the joining points near the sensor chamber. Sample 2 had an improved setup, with which oxygen could be pumped out, so that 5% could be established with only 2.5 mA.

However, due to its long diffusion paths, the initial demonstrator setup showed a prohibitively slow sensor kinetics. Furthermore, the hydrocarbons reacted at the walls and since only few hydrocarbons reached the film, the sensitivity of this initial setup was lower than expected.

The assembly of sensor and pumping cell in [15] has been further developed and is called sensor “platform” in the following. This paper deals with the new design of the platform and the integration of the two-sensor-setup. The catalytic activation of one of the sensor layers is discussed and sensors with Ga_2_O_3_ and 1% Nb-doped SrTiO_3_ as gas sensitive films have been tested. These are the pilot test of the new sensor platform. The main function of the platform, which is the detection of HCs under a defined oxygen atmosphere independent of the oxygen partial pressure in the exhaust gas, has to be investigated in the next step.

## 2. Experimental Section

### 2.1. Platform Design

**Figure 3.**
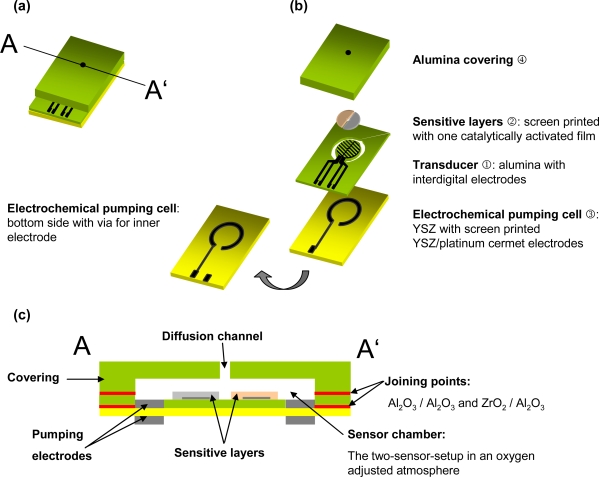
(a) Assembled sensor platform. (b) Exploded view. (c) Cross section.

**Figure 4.**
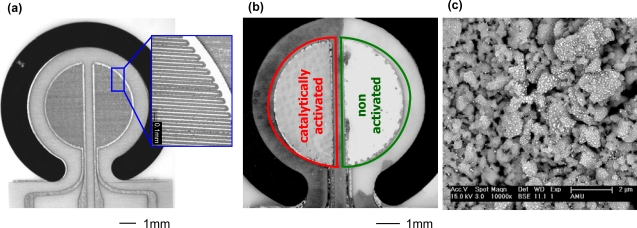
Transducer. **(a)** Two interdigital electrodes in the shape of a semi-circle are displayed. In the magnification, the laser-patterned electrodes are visible. **(b)** Screen-printed and sintered gas sensitive layers on the electrodes. The left layer has been activated. The grey color results from platinum clusters on the surface which provide the activation. **(c)** SEM image of the impregnated and thermally treated Ga_2_O_3_ sensor layer.

The sensor design can be seen in Figure 3. All bold highlighted terms are explained in the following. A transducer ① was made in High-Temperature-Cofired-Ceramic technology (HTCC). Two alumina tapes (Keral 99, Kerafol GmbH, 99% Al_2_O_3_) were laminated with 15 MPa at 70 °C for 10 minutes. The “ring” in the transducer (see Figure 3b and Figure 4a) was cut by a 355 nm Nd:YAG laser (arc lamp pumped with Q-switching in a LPKF Microline 350L system). After lamination and sintering at 1560 °C, 2 μm platinum were sputtered on one side of the alumina ceramic. Later, the electrodes for the two-sensor-setup in the form of interdigital electrodes (IDEs) with 20 μm line and space were laser-patterned, using laser parameters as explained in [17]. After a subsequent thermal treatment at 1000 °C to restore laser damages, the transducer ① was completed.

For the sensitive layers ②, screen-printable pastes either of Ga_2_O_3_ or of 1% Nb-doped SrTiO_3_ were prepared by mixing the oxide powder and an organic binder consisting of terpineol and ethyl cellulose. For the preparation of the Ga_2_O_3_ paste 99.999% pure Ga_2_O_3_ powder was calcinated at 1150 °C for 1 h and dry milled in a planetary ball mill using milling balls of zirconium dioxide to decrease grain size to approximately 1–2 μm. 1% Nb-doped SrTiO_3_ powder was prepared in mixed-oxide technique as described in [18] for La-doped SrTiO_3_.

The sensitive layers were then printed on the IDE structure, dried, sintered and activated (for activation see below).

An 8% yttrium doped zirconia (YSZ) substrate was used for the electrochemical pumping cell ③. The electrode material, a platinum/YSZ cermet (8 wt% Y_2_O_3_, Fraunhofer IKTS) was screen-printed on both sides of the substrate. The inner electrode is connected by a laser-drilled via. The electrochemical pumping cell was then fixed to the transducer with a commercial glass solder (Aremco 617; Figure 3c). The inner pumping electrode fits the cut ring at this joint and is thereby connected to the sensor chamber.

The covering ④ consists of an alumina substrate and has a cavity and a diffusion channel, which were milled and drilled, respectively, by laser. The cavity is located above the sensor and provides the volume, in which the oxygen concentration has to be adjusted. The exhaust gas enters the platform through the diffusion hole, located above the center of the circular gas sensitive area. The covering was joined to the transducer by a ceramic paste (QM42, DuPont). It should be noted here, that at this stage the sensor setup has to be heated externally to operation temperature in a small tube furnace. In the next step, a screen-printed platinum heater structure was included between the tapes. See results for the modeling and testing of the heater in Section 3.1.

### 2.2. Activation

One of the two sensitive films is activated by impregnation with 10% hexachloroplatinic (IV) acid similar to a procedure described in [20]. During heating up to operation temperature, the acid evaporates and catalytically active small platinum clusters are generated on the surface of the metal oxide grains.

In Figure 4a, the resulting uncovered IDE structure is shown, whereas Figure 4b depicts the same sensor after applying the screen-printed sensitive films. The successful activation of the left side can be seen by the changed color. The SEM picture (Figure 4c) shows an impregnated and thermally treated Ga_2_O_3_ sensor layer. The nanosized platinum clusters (white) are dispersed very well on the surface.

### 2.3. Measurements

The two-sensor-setup was heated in a tube furnace to 650 °C. The gas flow of 60 L/h was supplied by a gas mixture facility and saturated with water in a fritted wash-bottle. The total H_2_O content amounted to approx. 2.3%. 25 ppm up to 1,000 ppm of the test gas propane were added to a base gas mixture of N_2_ and 2% or 5% O_2_. The tests of the oxygen cross-sensitivity started with 10% O_2_ and were reduced down to 0.1% O_2_. The resistive sensor signals were either recorded with a Keithley 2700 Multimeter/Data Acquisition System or a Novocontrol Alpha-A High Performance Frequency Analyser. Simultaneously to the sensitivity investigations, a platinum heater to adjust the sensor platform to working temperature was developed and tested. The temperature distribution over the heater was measured with a thermo camera (Varioscan 3011 ST, InfraTec).

## 3. Results and Discussion

### 3.1. Heater

**Figure 5.**
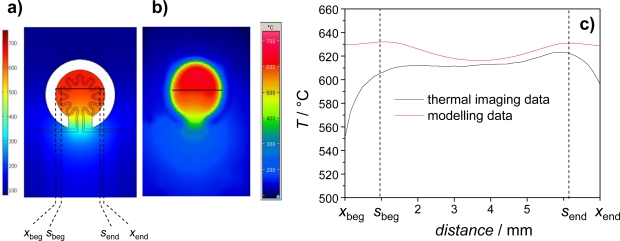
**(a)** Temperature distribution from simulation. **(b)** Measured temperature distribution. **(c)** Comparison between measurement and simulation along the indicated line.

In order to heat the sensor element itself, a buried heater was developed. Its design was modeled by using the commercial software Comsol Multiphysics. The software provides the possibility to couple different physical problems. In our case, heat transfer was coupled with electrical conductivity. Details of the calculation method (but for a completely different system) can be found in [19]. Figure 5a shows the calculated temperature distribution of the transducer with a rosette-like heater, which turned out (at as a first order approximation) to be the best design with respect to temperature homogeneity. The temperature distribution as obtained from a realized sensor (screen-printed platinum thick-film paste; LPA 88/11; line width 300 μm; laminated as described above) is depicted in Figure 5b. The temperature distribution was measured with the thermo camera. First of all, modeling and simulation agree quite well. Especially the calculated temperature in the area where the sensitive film is located (between *s*_beg_ and *s*_end_) is well simulated. For the application, it is important that the temperature distribution is homogenous over a wide range of the sensor area, which obviously is the case in both measurement and simulation.

### 3.2. Two-Sensor-Setup

**Figure 6.**
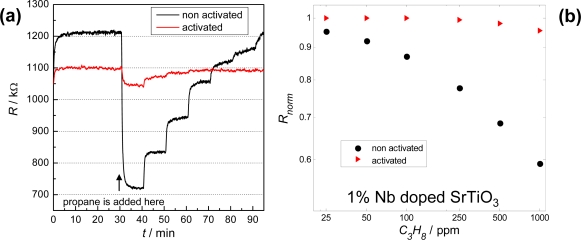
**(a)** Resistance signal *R* of catalytically activated and non-activated 1% Nb-doped SrTiO_3_ at humid base gas with 2% O_2_, 2.3% H_2_O in N_2_ and with 1,000, 500, 250, 100, 50 and 25 ppm propane, respectively. **(b)** Log-log plot of the normalized resistance, *R_norm_*, of the activated and the non-activated layer versus the propane concentration. *T* = 650 °C.

**Figure 7.**
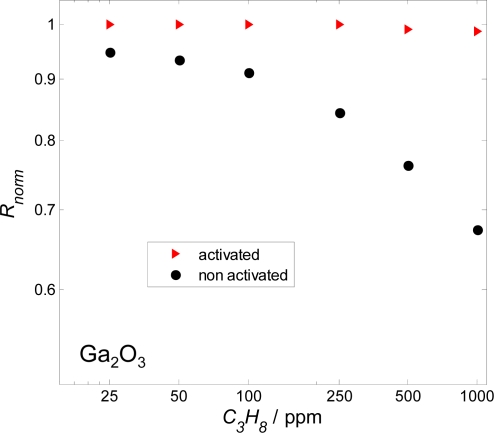
Log-log plot of the normalized resistance, *R_norm_*, of a Ga_2_O_3_ sensitive layer versus the propane concentration in 5% O_2_, 2.3% H_2_O, N_2_ balanced. *T* = 650 °C.

A typical measurement result of a two-sensor-setup is plotted in Figure 6a. The resistance signals of an activated and a non-activated 1% Nb-doped SrTiO_3_ layer are stable very soon after the measurement in the base gas has started. They also follow the propane concentration steps very quickly. The activated layer shows only a very slight reaction towards propane, whereas the non-activated sensor signal exhibits a resistance change up to 40%. This can be seen even better when the normalized resistance, *R_norm_*, (*R_norm_* = *R* / *R_0ppmHC_*) is plotted versus the concentration of the test gas propane (Figure 6b). Similar results can be observed for Ga_2_O_3_ (Figure 7). The non-activated layer is very sensitive towards propane, while the resistance of the activated one changes only marginally.

Both the activated and the non-activated layer should be sensitive towards oxygen. On the one hand, the idea of the two-sensor-setup depends on the oxygen cross-sensitivity and on the other hand, the signal of the activated layer should be used to control the oxygen current through the electrochemical pumping cell.

**Figure 8.**
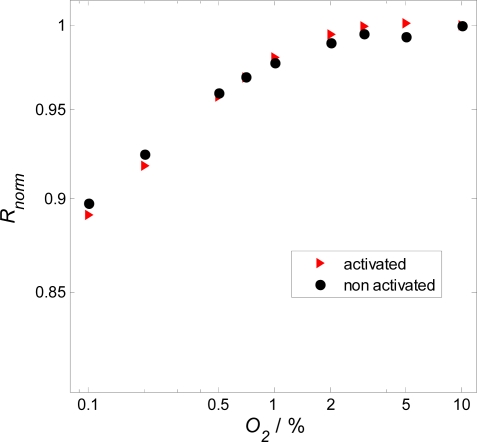
Log-log plot of the normalized resistance, *R_norm_* of 1% Nb-doped SrTiO_3_ versus the oxygen concentration in 2.3% H_2_O. *T* = 650 °C

In Figure 8, the dependency of the film resistance towards the oxygen concentration is displayed. The normalized resistance signal of activated and non-activated films behaves almost identical. Due to the 1% Nb-doping, the sensor is in the n-type region but reaches the so called intrinsic conductivity minimum at high oxygen concentrations of 10%. This result agrees completely with defect chemistry data of the literature [21].

Thus, two components of the sensor platform could be produced and tested. It was shown that it is possible to pattern platinum IDEs by laser. Secondly, the integration of the two-sensor-setup into the new platform design worked out. The tests showed that the sensor signal of activated and non activated sensor layer attained comparable results to [12].

It should be noted here that this platform may also be used for other types of high-temperature sensors that need a defined oxygen concentration for proper operation, i.e., for mixed potential sensors. Since all three components, the transducer, the electrochemical pumping cell, and the covering are prepared separately before being joined without prohibitively high temperature steps, a modular platform is achieved.

## 4. Conclusions

A new sensor platform has been introduced. Very often high-temperature sintering steps as applied in zirconia multilayer technology (like, e.g., for the planar λ-probes) are impossible. Since many sensor materials cannot be co-fired with YSZ due to their lower sintering temperature, methods of low temperature joining are used, e.g., YSZ and Al_2_O_3_ are joined with glass solders. Besides conventional technologies such as tape technology and screen-printing, the laser cutting and laser patterning technology was employed. It provided a great freedom of design.

As an exemplary application, a setup using two resistive sensors for HC detection was successfully integrated into the new platform. Activated and non-activated sensor films were tested successfully. On the sensor platform, it is therefore possible to eliminate the remaining oxygen cross-sensitivity of the HC signal by the quotient of the sensor signals of activated and non-activated sensor layer. In order to improve accuracy and to operate the sensor also in rich atmospheres, the electrochemical pumping cell adjusts the oxygen concentration at the sensitive film. A platform heater could be developed and also tested successfully.

In the next step the two-sensor-setup with an integrated heater has to be joined with the electrochemical pumping cell and the covering in order to present the platform which has been introduced here. Tests of HC detection under a defined oxygen concentration at the sensitive layers have to be conducted.
